# FUNCTIONAL LIMITATIONS AND MORTALITY AMONG PERSONS AGING WITH HIV INFECTION AND SUBSTANCE USE

**DOI:** 10.1093/geroni/igae098.4335

**Published:** 2024-12-31

**Authors:** Hema Ramamurthi, Ann Gruber-Baldini, Denise Orwig

**Affiliations:** Bloomberg School of Public Health, Johns Hopkins University, Baltimore, Maryland, United States; University of Maryland School of Medicine, Baltimore, Maryland, United States; University of Maryland School of Medicine, Baltimore, Maryland, United States

## Abstract

Aging individuals living with HIV experience earlier onset of adverse health outcomes, increased hospitalization rates, and mortality. However, there is limited research demonstrating the impact of HIV, substance use and functional limitations on survival. We analyzed self-reported longitudinal data collected during biannual visits from 2005 to 2020 among 2,633 participants contributing 120527 study visits in the community-engaged, AIDS Linked to the Intravenous Experience (ALIVE) cohort. Functional limitation was defined as having reported “limited a lot” in one of the 4 items of the physical activity section of Medical Outcomes Study-HIV scale. Scores from each visit were summed and categorized into: No Limitation = 0, Moderate Level of Limitation = 1-2 and Severe Limitation > 3. The sample included 751 (28.52%) People Living with HIV (PLWH), who were slightly older than HIV-negative counterparts (47.97 years vs. 44.89 years, p< 0.001). PLWH had a higher prevalence of functional limitations compared to HIV-negative individuals (44.1% vs. 38.3%, p< 0.05). In Cox Proportional Hazards models adjusted for socio-demographic, lifestyle factors and comorbidities, detectable viral load (HR=2.49, CI=2.08-2.97), and having 1-2 or >3 functional limitations (HR=1.27, CI =1.07-1.50 & HR=1.78, CI=1.51-2.10) respectively were predictive of mortality. This analysis highlights the importance of self-reported functional limitations and other potentially modifiable risk factors as important correlates of mortality among PLWH. These factors should be of key focus in the development of future interventions to effectively reduce adverse outcomes among PLWH.

